# Bone-patellar tendon-bone versus two- and four-strand hamstring tendon autografts for ACL reconstruction in young adults: a Bayesian network meta-analysis

**DOI:** 10.1038/s41598-023-33899-1

**Published:** 2023-04-27

**Authors:** Filippo Migliorini, Ernesto Torsiello, Andromahi Trivellas, Jörg Eschweiler, Frank Hildebrand, Nicola Maffulli

**Affiliations:** 1 Department of Orthopaedic, Trauma, and Reconstructive Surgery, RWTH University Hospital, Pauwelsstraße 30, 52074 Aachen, Germany; 2 Department of Medicine, Surgery and Dentistry, University of Salerno, Via S. Allende, 84081 Baronissi, SA Italy; 3 Department of Orthopaedic and Trauma Surgery, Geffen School of Medicine at University of California Los Angeles (UCLA), Los Angeles, 90095 USA; 4 School of Pharmacy and Bioengineering, Keele University Faculty of Medicine, Thornburrow Drive, Stoke on Trent, England; 5 Queen Mary University of London, Barts and the London School of Medicine and Dentistry, Centre for Sports and Exercise Medicine, Mile End Hospital, 275 Bancroft Road, London, E1 4DG England

**Keywords:** Health care, Medical research, Signs and symptoms

## Abstract

Bone-patellar tendon-bone (BPTB), two- and four-strand hamstring tendon (4SHT and 2SHT, respectively) are the most common autografts used for anterior cruciate ligament (ACL) reconstruction. The present study compared BPTB, 2SHT, and 4SHT for ACL reconstruction in terms of joint laxity, patient reported outcome measures (PROMs), rate of failure and anterior knee pain (AKP). The time to return to sport and the peak torque between the autografts were also compared. Finally, prognostic factors leading to worse outcomes were also investigated. It was hypothesized that all grafts yield similar proprieties in terms of joint laxity, patient reported outcome measures (PROMs) and rate of failure, but that the BPTB autograft causes a greater rate of anterior knee pain (AKP). The literature search was conducted. All clinical trials comparing BTPB and/or 2SHT, and/or 4SHT were accessed. Grafts other than BTPB and/or 4SHT and/or 2SHT were not considered. Articles reporting outcomes of allografts or synthetic grafts were not eligible, nor were those concerning revision settings. Articles reporting ACL reconstruction in patients with multi-ligament damage were also not eligible. Data from 95,575 procedures were retrieved. The median length of follow-up was 36 months. The median age of the patients was 27.5 years. With regard to joint laxity, similarity was found in terms of Lachman and Pivot shift tests between all three autografts. The BPTB demonstrated the greatest stability in terms of instrumental laxity. BPTB demonstrated the greatest PROMs. BPTB demonstrated the greatest rate of AKP, while AKP in 2SHT and 4SHT was similar. Concerning failure, statistically significant inconsistency was found (P = 0.008). The 4SHT demonstrated the quickest return to sport, followed by BPTB, and 2SHT. There was evidence of a negative association between the time span between injury to surgery, Lysholm score (P = 0.04), and Tegner scale (P = 0.04). Furthermore, there was evidence of a weak positive association between the time span between injury to surgery and return to sport (P = 0.01). BPTB may result in lower joint laxity, greater PROMs, and greater peak flexion torque compared to 2SHT and 4SHT autografts. On the other hand, BPTB reported the lowest peak extension torque and the greatest rate of AKP. Finally, a longer time span between injury and surgery negatively influences outcome.

## Introduction

The anterior cruciate ligament (ACL) is the primary passive constraint for internal tibial rotation and anterior tibial translation over the femur^1–4^. ACL injury is one of the most common knee injuries in the young athletic population^5,6^, most commonly in those performing jumping, twisting and cutting movements^7^. Its estimated incidence worldwide is about 70 per 100,000 people per year^8–12^. Anterior cruciate ligament rupture affects the knee kinematics^13–15^ resulting in joint instability, articular cartilage injury, and meniscal damage^14–27^. The optimal management of ACL is still debated^28,29^. Likewise, despite thousands of clinical articles on ACL surgical treatment, controversies still remain regarding the optimal choice of graft^30–34^. Bone-patellar tendon-bone (BPTB) and hamstring tendon (HT) autografts are the most common options for primary anterior cruciate ligament reconstruction^35,36^. The use of the BPTB autograft was introduced in the 1980s^37^ and it is still one of the most commonly used^38^. BPTB autografts achieve high patient satisfaction, quick return to sport and bone-to-bone healing^39,40^. However, concerns have been raised about donor site complications after BPTB autograft, such as anterior knee pain, discomfort, crepitus, loss of sensation, patellar fractures, contracture of the lower patella, and loss of extension strength^41–43^. To reduce damage to the extensor apparatus, the rates of anterior knee pain and patellar fractures, hamstring tendon (HT) autograft has been advocated^44–47^. However, ACL reconstruction using HT autograft may lead to a greater tunnel widening, flexor weakness, and knee laxity compared to BPTB^42,48,49^. In addition, the lack of bone block on the extremities of the HT graft may promote greater laxity leading to higher frequency of rupture^50^. Several clinical studies compare the autografts mentioned above, but the results are inconclusive^35,51^. In this Bayesian network meta-analysis, BPTB, two- and four-strand HT (4SHT and 2SHT, respectively) autografts for ACL reconstruction in young adults were compared. Joint laxity, patient reported outcome measures (PROMs), rate of failure, and anterior knee pain (AKP) between the autografts were compared, as were the time to return to sport and the peak torque. A multivariate analysis was conducted to investigate possible prognostic factors leading to worse outcomes. It was hypothesized that all grafts yield similar proprieties in terms of joint laxity, PROMs, and rate of failure, but that the BPTB autograft causes a greater rate of anterior knee pain (AKP).

## Material and methods

### Search strategy

The present Bayesian network meta-analysis followed the Preferred Reporting Items for Systematic Reviews and Meta-Analyses (PRISMA) extension statement for reporting of systematic review incorporating network meta-analyses of health care interventions^52^. A PICO guide protocol was preliminary drafted:

- P (population): ACL tears in young adults;
- I (intervention): primary ACL reconstruction;
- C (comparison): BPTB, 4SHT, 2SHT;
- (outcomes): laxity, PROMs, failure, AKP.

### Data source and extraction

Two reviewers (**;**) separately performed the literature search in February 2023. PubMed, Google scholar, Embase, and Scopus databases were accessed. The following keywords were used using the Boolean operator AND/OR: *anterior cruciate ligament, ACL, pain, knee, tear, rupture, injury, damage, reconstruction, management, treatment, arthroscopy, surgery, autografts, bone patellar tendon bone, hamstring, strands, patient reported outcome measures, PROMs, laxity, stability, instability, complication, anterior knee pain, failure*. The resulting titles were screened by the same authors independently. If the title and the abstract matched the topic, the article’s full-text was accessed. If the full-text was not accessible, the article was excluded from the present study. A cross reference of the bibliographies was also performed. Disagreements were debated and the final decision was made by a third author (**).

### Eligibility criteria

All clinical investigations comparing BTPB, and/or 4SHT, and/or 2SHT were accessed. Articles in English, German, Italian, French, and Spanish were eligible. Levels I to III of evidence, according to Oxford Centre of Evidence-Based Medicine (OCEBM)^53^, were considered. Grafts other than BTPB and/or 4SHT and/or 2SHT were not eligible. Studies which reported data on skeletally immature patients were not considered. Articles reporting outcomes from allograft or synthetic graft reconstructions were not eligible, nor where those concerning revision settings. Articles reporting ACL reconstruction in patients with multi-ligament damage were not eligible. Letters, comments, reviews, opinions, and editorials were not included. Animals and biomechanics studies were also not considered. Only articles reporting quantitative data under the outcomes of interest were considered for inclusion. Missing data under the outcomes of interest warranted the exclusion from this study.

### Data extraction

Two authors (**;**) independently examined the resulting articles for inclusion. Generalities and patient demographic were retrieved: author, year, journal, study design, length of the follow-up, type of graft, number of included patients, mean age, BMI, sex, time span from injury to surgery, and size of the graft. To investigate knee stability, data from the manual (Pivot shift and Lachman tests) and instrumental laxity were extracted. The instrumental laxity was evaluated using the arthrometers KT-1000 and KT-2000 (MEDmetric Corp, San Diego, California). Both of these devices applied a force of 134N on the tibial plateau over the femoral condyles, directed anteriorly. Concerning PROMs, data from the Tegner activity scale and Lysholm score at the last follow-up were extracted. The Lysholm score and Tegner activity scale have been validated for knee ligament surgery^54–56^. Data concerning the peak torque and the return to sport were also retrieved. The rates of failure and AKP were also investigated.

### Methodology quality assessment

The methodological quality assessment was made using the risk of bias graph of the Review Manager Software (The Nordic Cochrane Collaboration, Copenhagen). The following risks of bias were evaluated: selection, detection, reporting, attrition, and other sources of bias.

### Statistical analysis

The statistical analyses were performed by the main author (FM) using STATA Software/MP, Version 14.1 (StataCorporation, College Station, Texas, USA). For descriptive statistics, mean and standard deviation were calculated. The analysis of variance (ANOVA) was performed to evaluate the baseline comparability, with P values > 0.1 considered satisfactory.

To assess the return to sport, the ANOVA test with Tukey post-hoc test and honestly significant difference (HSD) were performed, with values of P < 0.05 were considered statistically significant. The confidence interval (CI) was set at 95%.

The NMA was performed through the STATA routine for Bayesian hierarchical random-effects model analysis. The inverse variance method was used for analysis of continuous variable, with standardized mean difference (STD) effect measure. The Log odd ratio (LOR) effect measure was used for binary data. The overall inconsistency was evaluated through the equation for global linearity via the Wald test. If the P value was > 0.5, the null hypothesis could not be rejected, and the consistency assumption could be accepted at the overall level of each treatment. Both confidence (CI) and percentile (PrI) intervals were set at 95%. Edge plot, interval plots, and funnel plots were obtained and evaluated.

For the multivariate analysis, a multiple linear model regression with Pearson Product-Moment Correlation Coefficient (*r*) was used to establish whether patient characteristics (age, BMI, women, time from injury to surgery, and graft size) are associated with the outcome (Pivot shift and Lachman tests, instrumental laxity, Lysholm score, Tegner scale, return to sport, failure, and anterior knee pain). The Cauchy–Schwarz formula was used for inequality: + 1 was considered as positive linear correlation, while − 1 was a negative one. Values of 0.1 < |$r$| < 0.3, 0.3 < |$r$ | < 0.5, and |$r$ | > 0.5 were considered to have respectively small, medium, and strong association. The overall significance was assessed through the χ^2^ test, with values of P < 0.05 considered statistically significant.

### Ethical approval

This study complies with ethical standards.

## Results

### Search result

**Figure 1:**
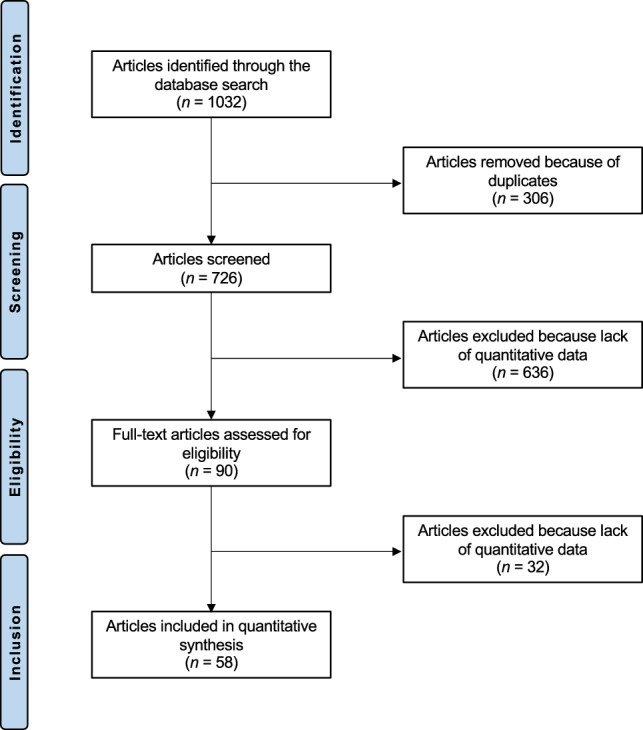
Flow chart of the literature search.

The literature search resulted in 1035 articles. Of them, 306 were excluded as they were duplicates. Furthermore, 636 articles were not eligible: not matching the topic (N = 403), reporting data on allografts or synthetic grafts (N = 41), study type (N = 154), revision or multi-ligament settings (N = 37), language limitation (N = 1). Additionally, 32 articles were excluded as they did not report quantitative data under the outcomes of interest. This left 61 clinical trials for the present study. The literature search results are shown in Fig. 1.

### Methodological quality assessment

**Figure 2:**
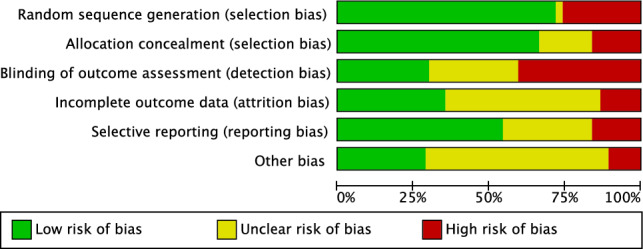
Methodological quality assessment.

The prospective design of 85% (52 of 61) of the included investigations was an important strength of the present study. Of them, 62% (32 of 52) performed randomisation. Since most of the studies performed assessor blinding, the risk of detection bias was moderate to low. The proper analyses of most of the included studies, along with the intention to treat, clear definition of the timing of assessing outcomes, as well the use of validated tools for assessing outcomes, lead to a low risk of reporting and attrition bias. The risk of other biases was moderate to low. In conclusion, the methodological quality assessment demonstrated a moderate to low risk of bias (Fig. 2).

### Patient demographics

Data from 102,573 procedures were retrieved. The median length of follow-up was 51.5 ± 49.4 months. The median age of the patients was 27.9 ± 4.2 years. The median time span from injury to surgery was 14.4 ± 11.2 months. The mean BMI was 24.6 ± 1.6. The median size of the graft was 9.7 ± 0.7 mm. The ANOVA test found moderate baseline comparability among age, length of the follow-up, time span from injury to surgery, BMI, and graft size (P > 0.05). Patient demographics is shown in Table 1.

**Table 1:** Generalities and patient baselines from the included studies.

Author, year	Journal	Study design	Follow-up (months)	Type of graft	Patients	Mean Age	Female (%)
Aglietti et al. 1994^57^	*Am. J. Sports Med.*	Prospective	28	BPTB	30		
				4SHT	30		
Aglietti et al. 2004^58^	*J. Bone Jt. Surg. Am.*	RCT	24	BPTB	60	25	23.00
				4SHT	60	25	23.00
Aune et al. 2001^45^	*Am. J. Sports Med.*	RCT	24	BPTB	35	25	45.71
				4SHT	37	27	43.23
Barenius et al. 2010^59^	*Am. J. Sports Med.*	RCT	100.8	BPTB	84	33	57.30
				4SHT	80	35	42.70
Beynnon et al. 2002^60^	*J. Bone Jt. Surg. Am.*	RCT	36	BPTB	28	28.5	35.71
				2SHT	28	29.9	53.57
Biz et al. 2019^61^	*Acta Biomed*	Prospective	44.8	BPTB	22	31.9	
				4 SH	21	31	
Bizzini et al. 2006^62^	*Clin J Sport Med*	Prospective	11	BPTB	87	34	37.90
				4SHT	66	31.3	31.82
Carter et al. 1999^63^	*Arthroscopy*	RCT	6	BPTB	38		
				2SHT	35		
Corry et al. 1999^64^	*Am. J. Sports Med.*	Prospective	24	BPTB	90	25	47.00
				4SHT	90	25	48.00
Cristiani et al. 2018^65^	*Knee Surg. Sports Traumatol. Arthrosc.*	Retrospective	12	BPTB	692	28.8	28
				4 SH	4770	28.1	46.5
Denti et al. 2006^66^	*Knee Surg. Sports Traumatol. Arthrosc.*	Prospective	24	BPTB	39	23.5	15.39
				2SHT	22	40.1	68.20
Drogset et al. 2009^67^	*Knee Surg. Sports Traumatol. Arthrosc.*	RCT	24	BPTB	58	26	
				2SHT	57	27	
Feller et al. 2001^68^	*Knee Surg. Sports Traumatol. Arthrosc.*	RCT	4	BPTB	31	26.2	25.81
				2SHT	34	27.1	29.41
Gifstad et al. 2013^35^	*Knee Surg. Sports Traumatol. Arthrosc.*	RCT	84	BPTB	58	27	
				4SHT	56	27	
Gifstad et al. 2014^69^	*Am. J. Sports Med.*	Prospective	48	BPTB	6736	29	37.10
				4SHT	38,666	26	42.80
Gobbi et al. 2003^39^	*Arthroscopy*	Prospective	36	BPTB	40	28	35.00
				4SHT	40	29	45.00
Gobbi et al. 2006^70^	*Knee Surg. Sports Traumatol. Arthrosc.*	RCT	24	BPTB	50	28	
				4SHT	50	28	
Gudas et al. 2018^71^	*Med. Sci. Monit.*	Retrospective	24	BPTB	88	26	30.68
				4SHT	95	25.1	30.53
Guglielmetti et al. 2021^72^	*Orthop J Sports Med*	Prospective Randomized	24	PT	31	25.2	26
				4 SH	31	24.64	39
Harilainen et al. 2006^73^	*Knee Surg. Sports Traumatol. Arthrosc.*	RCT	60	BPTB	40		
				2SHT	39		
Heijne et al. 2009^74^	*Knee Surg. Sports Traumatol. Arthrosc.*	RCT	24.7	BPTB	34	29	35.29
				4SHT	34	30	58.82
Heijne et al. 2013^75^	*Knee Surg. Sports Traumatol. Arthrosc.*	RCT	61.5	BPTB	34	29	35.29
				4SHT	34	30	58.82
Holm et al. 2010^76^	*Am. J. Sports Med.*	RCT	120	BPTB	28	25	35.17
				4SHT	29	27	48.27
Ibrahim et al. 2005^77^	*Arthroscopy*	RCT	81	BPTB	40	22.3	0
				4SHT	45	22.3	0
Jansson et al. 2003^78^	*Am. J. Sports Med.*	RCT	21	BPTB	43		
				4SHT	46		
Kautzner et al. 2014^79^	*Int Orthop*	RCT	24	BPTB	75	26	
				4SHT	75	26	
Keays et al. 2007^80^	*Am. J. Sports Med.*	Retrospective	72	BPTB	31	27	29.00
				4SHT	31	27	29.00
Laxdal et al. 2006^81^	*Knee Surg. Sports Traumatol. Arthrosc.*	Prospective	25	BPTB	45	26	0.00
				4SHT	78	28	0.00
Leitgeb et al. 2014^82^	*Wien Klin Wochenschr*	RCT	60	BPTB	56	28.4	19.64
				4SHT	40	29.2	42.50
Leys et al. 2011^83^	*Am. J. Sports Med.*	Prospective	180	BPTB	90	25	46.67
				4SHT	90	24	47.78
Lidén et al. 2007^84^	*Am. J. Sports Med.*	RCT	86	BPTB	34	28	32.35
				4SHT	37	29	29.73
Machado et al. 2018^85^	*Phys Sportsmed*	RCT	6	BPTB	17	31.9	29.41
				4SHT	17	37.7	17.65
Maletis et al. 2007^86^	*Am. J. Sports Med.*	RCT	24	BPTB	46	27.2	32.61
				4SHT	53	27.7	15.09
Maletis et al. 2013^87^	*Bone Joint J*	Retrospective	18	BPTB	2791	25.4	30.60
				4SHT	3012	27.2	39.70
Marder et al. 1991^88^	*Am. J. Sports Med.*	Prospective	29	BPTB	37	21.6	35.14
				4SHT	35	23.8	25.71
				4SHT	23	18	57.00
Matsumoto et al. 2006^51^	*Am. J. Sports Med.*	RCT	80	BPTB	37	23.7	43.24
				4SHT	35	24.4	57.14
Mohtadi et al. 2015^89^	*Clin J Sport Med*	RCT	24	BPTB	110	28.7	42.72
				4SHT	110	28.5	46.36
Pasquini et al. 2017^90^	*Acta Biomater*	Prospective		BPTB	15	26	0.00
				4SHT	15	30.3	0.00
Persson et al. 2013^91^	*Am. J. Sports Med.*	Retrospective	48	BPTB	3428	29	41.10
				4SHT	9215	28.3	43.20
Persson et al. 2015^92^	*Am. J. Sports Med.*	Retrospective	54	BPTB	3806	28.8	41.70
				4SHT	10,228	28.36	44.08
Pinczewski et al. 2007^93^	*Am. J. Sports Med.*	Prospective	120	BPTB	90		
				4SHT	90		
Pinczewski et al. 2016^94^	*Am. J. Sports Med.*	Prospective	60	BPTB	90	25	47.00
				4SHT	90	24	48.00
Predescu et al. 2010^95^	*IEEE CS*	Prospective	12	BPTB	76		
				4SHT	59		
Rahr-Wagner et al. 2013^96^	*Am. J. Sports Med.*	Prospective	36	BPTB	1971		34.00
				4SHT	11,676		42.00
Razi et al. 2014^97^	*Med J Islam Repub Iran*	RCT	36	BPTB	37	30.8	21.62
				4SHT	34	28.2	14.71
Sadoghi et al. 2011^98^	*Int Orthop*	Prospective	24	BPTB	41	30	
				4SHT	51	29	
Sajovic et al. 2006^99^	*Am. J. Sports Med.*	RCT	60	BPTB	32	27	46.15
				4SHT	32	24	53.57
Sajovic et al. 2011^100^	*Am. J. Sports Med.*	RCT	132	BPTB	32	38	36.00
				4SHT	32	36	48.15
Sajovic et al. 2018^101^	*Am. J. Sports Med.*	RCT	204	BPTB	32	45.5	37.50
				4SHT	32	42.5	45.83
Shaieb et al. 2002^47^	*Am. J. Sports Med.*	RCT	24	BPTB	33	32	21.21
				4SHT	37	30	43.24
Stanczak et al. 2017^102^	*J Int Med Res*	RCT	12	BPTB	48	31.6	16.66
				4SHT	48	31.6	25.00
Svensson et al. 2005^103^	*Knee Surg. Sports Traumatol. Arthrosc.*	Prospective	24	BPTB	28	28	100.00
				4SHT	31	25	100.00
Tajima et al. 2020^104^	*J Knee Surg*	Prospective	26	BPTB-G	32	22.7	93.75
				2 SH	43	24.8	93
Taylor et al. 2009^105^	*Am. J. Sports Med.*	RCT	36	BPTB	32	21.7	21.90
				4SHT	32	22.1	12.50
Thompson et al. 2016^106^	*Am. J. Sports Med.*	Prospective	240	BPTB	90	25	46.67
				4SHT	90	24	47.78
Wagner et al. 2005^107^	*Am. J. Sports Med.*	Prospective	24	BPTB	55	33.6	27.27
				4SHT	55	31.1	27.27
Webster et al. 2015^108^	*Am. J. Sports Med.*	RCT	180	BPTB	22	26.6	27.27
				4SHT	25	26.1	20.00
Wipfler et al. 2011^109^	*Arthroscopy*	RCT	105	BPTB	31	29.87	38.71
				4SHT	31	34.23	41.94
Witvrouw et al. 2001^110^	*Int Orthop*	Prospective	12	BPTB	17	24.3	41.18
				4SHT	32	24.6	46.88
Zaffagnini et al. 2006^111^	*Knee Surg. Sports Traumatol. Arthrosc.*	RCT	60	BPTB	25	30.5	36.00
				4SHT	25	31.3	40.00
Zoran et al. 2015^112^	*Inj Epidemiology*	Prospective	24	BPTB	54	28	27.70
				4SHT	58	26	27.70

### Network comparisons

**Figure 3:**
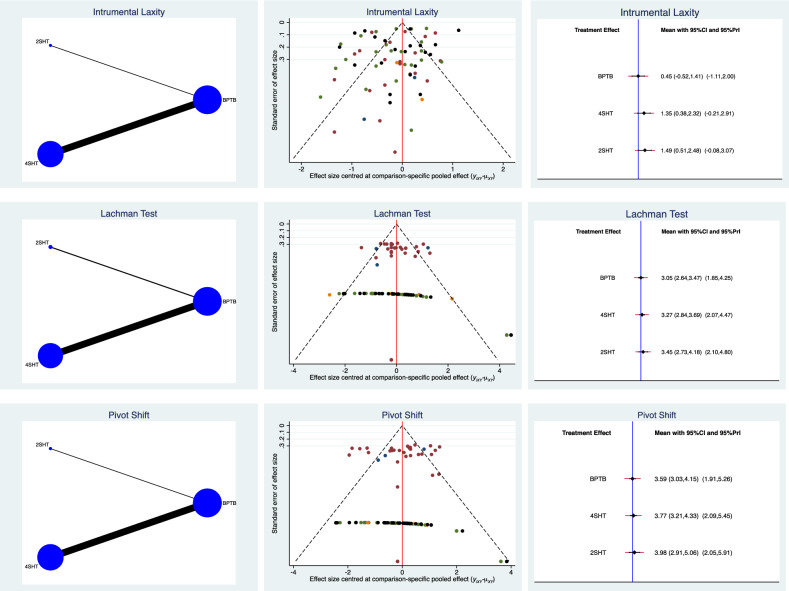
Edge, funnel, and interval plots of the network comparisons: joint laxity.

With regard to joint laxity, similarities were found in terms of Lachman and Pivot shift tests between all three autografts. The BPTB demonstrated the greatest stability in terms of instrumental laxity. The equation for global linearity found no statically significant inconsistency (P = 0.06, P = 0.08, and P = 0.1, respectively). These results are shown in greater detail in Fig. 3.

**Figure 4:**
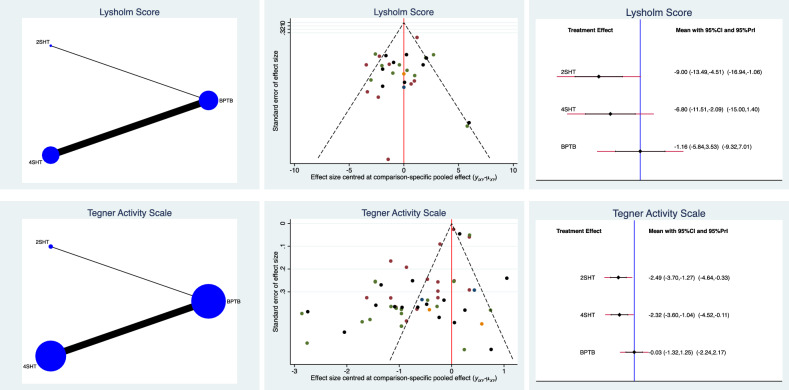
Edge, funnel, and interval plots of the network comparisons: PROMs.

Concerning PROMs, BPTB demonstrated the greatest Lysholm score and Tegner activity scale, followed by 2SHT and 4SHT, which scored similarly (Fig. 4). The equation for global linearity found no statically significant inconsistency (P = 0.3 and P = 0.5, respectively).

**Figure 5:**
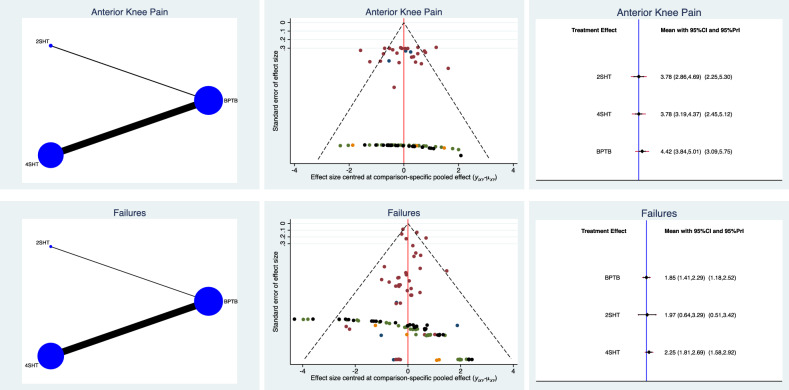
Edge, funnel, and interval plots of the network comparisons: complication.

Patients who underwent reconstruction of the ACL using a BPTB graft demonstrated the greatest rate of anterior knee pain, while both 2SHT and 4SHT ranked similarly. No statistically significant inconsistency was found (P = 0.2). The equation for global linearity found statistically significant inconsistency for the comparison of graft failure (P = 0.008), thus no further conclusion could be inferred. The network comparisons of complications are shown in greater detail in Fig. 5.

### Peak torque

Given the lack of quantitative data concerning the 2SHT group, only BPTB and 4SHT were considered for analysis of peak torque. BPTB demonstrated greater peak flexion torque at 60° (P < 0.0001) and 180° (P < 0.0001). No difference was found at 120° (P = 0.06). BPTB demonstrated lower peak extension torque at 60° (P = 0.01), 120° (P = 0.008), and 180° (P = 0.006). These results are shown in greater detail in Table 2.

**Table 2:** Peak torque.

Variables	4SHT	BPTB	MD	95% CI	P
Peak flexion torque 60° (deg/sec)	93.2 ± 6.2	99.1 ± 1.5	5.9	− 7.1 to − 4.6	< 0.0001
Peak flexion torque 120° (deg/s)	97.9 ± 2.8	98.8 ± 3.9	0.9	− 1.8 to 0.1	0.06
Peak flexion torque 180° (deg/s)	94.3 ± 4.9	100.3 ± 3.0	6.0	− 7.1 to − 4.8	< 0.0001
Peak extension torque 60° (deg/s)	93.0 ± 6.7	77.0 ± 31.3	− 16.0	9.6 to 22.3	0.01
Peak extension torque 120° (deg/s)	98.2 ± 3.2	96.4 ± 2.9	− 1.8	0.9 to 2.6	0.008
Peak extension torque 180° (deg/s)	96.2 ± 4.2	94.3 ± 5.4	− 1.9	0.5 to 3.2	0.006

### Return to sport

The 4SHT demonstrated the quickest return to sport, followed by BPTB, and 2SHT (Table 3).

**Table 3:** Return to sport.

	2SHT	4SHT	BPTB
2SHT	1		
4SHT	MD − 1.1; 95% CI − 1.45 to − 0.74; P < 0.0001	1	
BPTB	MD − 0.9; 95% CI − 1.25 to − 0.54; P < 0.0001	MD 0.2; 95% CI 0.03–0.36; P = 0.01	1

### Multivariate analysis

There was evidence of a negative association between the time span between injury to surgery and Lysholm score (*r* = − 0.50; P = 0.04) and Tegner scale (*r* = − 0.26; P = 0.04). Furthermore, there was evidence of a weakly positive association between the time span between injury to surgery and return to sport (*r* = − 0.06; P = 0.01). The results of the multivariate analysis are shown in Table 4.

**Table 4:** Results of the multivariate analysis (AKP = Anterior knee pain).

Endpoints	Age		BMI		Women		Time: injury to surgery		Graft size	P
	*r*	P	*r*	P	*r*	P	*r*	P	*r*	
Pivot shift test	− 0.14	0.08	− 0.21	0.8	− 0.22	0.1	0.28	0.04	− 0.24	0.6
Instrumental laxity	0.13	0.7	− 0.02	0.8	− 0.09	0.3	0.00	0.00002	− 0.14	0.5
Lachman test	0.11	0.3	− 0.80	1.0	− 0.40	0.5	− 0.48	1.0	0.00	0.6
Lysholm score	0.18	0.5	− 0.89	0.5	0.31	0.2	− 0.50	0.04	− 0.30	1.0
Tegner scale	0.13	0.4	− 1.00	0.05	− 0.40	0.26	− 0.26	0.04	− 0.73	0.4
Return to sport	− 0.04	0.6	0.09	0.3	0.06	0.5	0.06	0.01	− 0.35	0.2
Failure	0.33	0.7	− 0.06	0.7	0.04	0.6	0.17	0.6	0.12	0.07
AKP	0.00	0.02	0.58	0.9	0.67	0.8	0.74	0.3	0.86	0.6

## Discussion

According to the main findings of the present study, BPTB may promote lower joint laxity, greater PROMs, and greater peak flexion torque compared to 2SHT and 4SHT autografts in young adults. The ACL is one of the most important constraints against anteroposterior translation of the knee^113,114^. In the present study, BPTB was associated with the lowest peak extension torque and the greatest rate of AKP. Peak flexion torque is used to assess knee flexor muscle strength after reconstruction, as a quantitative outcome measure, particularly when comparing hamstring autografts to alternative graft options. Knee flexor weakness in knee flexion is relevant in certain sports such as gymnastics, judo, or wrestling, and it is useful to assess the return to sport^115^. Knee torque is significantly affected after ACL injury. Both extension and flexion isokinetic strength are important outcomes to evaluate after surgical reconstruction^116^. AKP remains a major complication after ACL reconstruction, and potentially recognizes several aetiologies, including bone-harvesting pain, neuroma of the infrapatellar branch of the medial saphenous following its lesion, and rarely, patellar tendinopathy^33^. Finally, a longer time span between ACL rupture and reconstruction may represent a negative factor influencing the outcome.

Concerning joint laxity, similarity was found in terms of Lachman and Pivot shift tests between all three autografts. The Lachman test evaluates the anterior translation of the tibia in relation to the femur with the knee in static flexion^117^. The Pivot shift test instead assesses the rotatory instability of the joint during its dynamic flexion^118^. Similarly, a previous meta-analysis found no difference in IKDC score, Lachman and Pivot shift tests between BPTB and hamstring autografts^119^. However, BPTB autograft resulted in a higher incidence of AKP, kneeling pain, and rate of osteoarthritis^119^. The literature on osteoarthritis of patients undergoing reconstruction with BPTB or HT autografts is controversial^120,121^. In the present study, patients receiving a BPTB graft demonstrated the lowest instrumental laxity and the greatest Lysholm score and Tegner activity scale, followed by 2SHT and 4SHT, which scored similarly. The Lysholm score and Tegner activity scale are outcomes measurements of a subjective nature that evaluate performance and activity restrictions both before and after surgery^122^. These PROMs have been validated for knee ligament surgery^54–56^.

On the other hand, BPTB demonstrated the greatest rate of AKP compared to both the 2SHT and 4SHT autografts, which showed a similar rate. Concerning failure, no statistically significant inconsistency was found. The equation for global linearity found statistically significant inconsistency for the comparison failure; thus, no further conclusion could be inferred. In a study on 5462 patients with primary ACL reconstruction, HT autografts resulted in greater anterior knee laxity and failures compared with BPTB autografts^65^. In a previous meta-analysis including 25 studies (47,613 ACL reconstructions), HT autografts failed at a higher rate than BPTB autografts^123^. Similar results have been evidenced in another meta-analysis involving 15 RCTs (1298 patients)^121^. A further meta-analysis including 20 RCTs compared BPTB versus 4SHT. The BPTP cohort evidenced lower laxity and failure rupture, but a greater risk of kneeling pain and AKP^124^.

Given the lack of quantitative data concerning the 2SHT group, only BPTB and 4SHT were considered in our study for analysis of peak torque. BPTB demonstrated greater peak flexion torque at 60° and 180°. No difference was found at 120°. BPTB demonstrated lower peak extension torque at 60°, 120°, and 180°. While BPTB exhibits some better outcome measures, it should be noted that BPTB also demonstrated the greatest rate of AKP. These findings agreed with previous studies comparing HT and BPTB, which stated that the latter restores greater knee stability, but also results in greater postoperative complications^121,125,126^. AKP is common following ACL reconstruction and can persist for a long time in athletes. The removal of the central third of the patellar tendon and its subsequent repair might cause a lowering of the patella and lead to increased sensitivity and pain during kneeling or squatting^127^. In this regard, in our study, the 4SHT graft demonstrated the quickest return to sport, followed by BPTB, and 2SHT. This should be considered when making a decision with athletes whose goal is to return to play as soon as possible. Lastly, results from the multivariate analysis demonstrated that a longer time span between initial injury and surgery was associated with lower Lysholm scores, Tegner scale, and longer return to sport. This worse outcome associated with a longer time from injury to surgery should be considered when planning the reconstruction. It should also be noted that some insurance companies currently require a dedicated physiotherapy trial for ACL injuries before surgery is authorized^128^. This delay in treatment can lead to suboptimal results^129,130^.

This study has certainly limitations. The retrospective nature of most studies is an important limitation which increases the risk of selection bias. Demographic data of the patients were collected, but further information regarding their general health were seldom reported in the included studies. Most of the authors did not specify whether the surgeon who performed the procedure was the investigator himself, and whether the assessor was blinded to the procedure performed. Many studies did not clearly specify the surgical technique (arthroscopic, open, or both) or postoperative management. Rehabilitation protocols following ACL reconstruction are associated with significant differences in outcome^131^. Several new modalities of rehabilitation after ACL reconstruction such as strengthening, and functional exercises, resistance training, neuromuscular exercise, high-level dynamic functional tasks and sport-specific training have been proposed^132–136^. However, given the lack of quantitative data, the various rehabilitation protocols could not be analysed separately. Most authors did not specify whether patients had undergone MRI preoperatively, thus providing poor information on preoperative diagnostic methods. Most authors did not report information on the sporting activity and level of the patients; therefore, further subgroup analyses were not possible. Given the lack of quantitative data, it was not possible to investigate additional autografts^137,138^. Allografts have been advocated as they avoid donor site morbidity^139–141^. However, the greater risk of graft-versus-host reaction, disease transmission, and delayed graft incorporation limits the use of allografts^142–144^. There is also a growing trend of using quadriceps tendon grafts, which may provide another viable and safe alternative for autografts options^145–148^. This autograft may result in a lower rate of failure compared to both BPTB and HT grafts, as well as a reduced rate of AKP compared to the BPTB autograft^149^. Further high-quality investigations should validate the present results also in skeletally immature patients. Furthermore, the aetiology of the AKP following ACL surgery still remains debated, and international recommendations on the management and classification of this condition are required.

## Conclusion

BPTB may promote lower joint laxity, greater PROMs, and greater peak flexion torque compared to 2SHT and 4SHT autografts. On the other hand, BPTB resulted in the lowest peak knee extension torque and the greatest rate of AKP. Concerning PROMs and AKP, similar scores were obtained in the comparison between SHT2 and 4SHT. However, the 4SHT demonstrated the quickest return to sport, followed by BPTB, and 2SHT. Finally, longer time span between injury and ACL reconstruction negatively influences the outcomes.

## Data Availability

The datasets generated during and/or analysed during the current study are available throughout the manuscript.
